# Memoirs upon the Tumors of the Gums, Known under the Name of Epulis

**Published:** 1858-01

**Authors:** L. Saurel


### ARTICLE VII.

*Memoirs upon the Tumors of the Gums, known under the name of Epulis.* By DR. L. SAUREL. Translated from the French for this Journal.

Two patients attacked with tumors of the gums having come under my observation during the years 1853 and 1856, I have naturally been led to make some researches into the nature and treatment of these tumors.

Although these diseases have been long known and appear not to be very rare, the works on surgery and the periodicals contain but a very small number of examples. I will use, to the best of my ability, those which I possess, to trace a monograph of *epulis.*

1. *History.** Ambrose Paré is perhaps the earliest writer who has left us a moderately exact description of epulis. The chapter which he has devoted to this malady, as remarkable for its exactness as for its conciseness, deserves to be reproduced entire.

* I must apprise the reader, that I do not pretend to have cited all the writers who have treated of epulis. I have consulted all that I have at my disposal, and in whose writings I thought I could find anything pertaining to this subject. There may be others, but I do not think they can be of great importance.

"Epulis," says he, "is a fleshy excrescence, which is formed upon the gums between the teeth, which gradually increases, sometimes being as large as an egg or larger, so that it interferes with talking and eating, emitting a salivary moisture of fetid odor; often it becomes cancerous, and then it cannot be touched; a condition which can be recognized by the heat, pain and other accidents. That which is not painful can be extirpated, an operation accomplished by tying it with a double thread and tightening the ligature till the tumor falls; after which, to prevent its return, the root must be cauterized, with a cautery in a canula hereafter described, or with a potential cautery, as oil of vitriol or aquafòrtis, if properly applied, so as to do no injury to the sound parts. I have amputated some which were so large, that portions of them projected beyond the cavity of the mouth, which rendered the patient hideous to look upon, and never had surgeon dared to undertake a cure, because the said excrescence was of a livid color, and I considered, setting aside this lividity, that it had little or no feeling: then I got courage to cut it, then to cauterize, and the patient was entirely cured, not always at first, but after repeated operations, because it grew again although it had been cauterized. The cause of this was a small portion of the bone of the alveolus, into which teeth are inserted, which was diseased and rotten. I have found some, which, in a long space of time, had degenerated into cartilage and even into bone; and therefore we should attempt the cure as early as possible. For when they are small and not rooted deeply, they are easier to cure; while we often find a glairy humor within, which gradually hardens and renders them very difficult to cure."

It is impossible to give in a few lines a more exact description of the malady which occupies our attention. In fact, almost all the species or varieties of epulis admitted by authors of our days are here specified, as well as the danger which some of them bring. But one thing can be questioned, that is the advice not to touch those which are cancerous; but in that Paré only shared an opinion generally entertained by his predecessors and cotemporaries; we cannot, therefore, seriously reproach him with it.

The *Memoirs of the Academy of Surgery*, which contain so many interesting researches, have but one memoir relating to epulis. This article, entitled, "Fungous excrescence of the gums," is composed of six records of cases, five of which are borrowed from ancient authors, and all leaving much to be desired in reference to the nature and structure of the tumors. The doctrine of Ambrose Paré relative to treatment, is adopted by the Academy of Surgery.

Before and since that time, several authors, as Mauget, Jourdain, Desault, &c., have related cases of tumors of the gums, but without making a special study of them.

An author whose work deserves to be much more read and consulted than it really is, Léveillé, has given under the title of *osteo sarcoma, or fungus of the gums,* a description of the tumors of the gums, from which I extract a few passages.

"A red excrescence," says he, "projecting upon the membrane which clothes the alveolus, is indolent, immovable, rarely soft and fungous, oftener hard, resisting, callous or cartilaginous. Seldom does it degenerate into cancer, undoubtedly, because the patient succumbs too early under his sufferings. This tumor is always accompanied by alteration of the bone, in the parenchyma of which it has its seat. The opening of bodies show only a homogeneous, hard, lardaceous substance, common to the gums, the cellular tissue, the muscles, the bones, and in which the teeth hardly visible by their crowns, are movable, vascillating and hardly carious. This autopsy leads us to conclude that the primitive seat of this organic lesion is in the parenchyma of the bone, the nutritive functions of which are no longer regular, and in which calcareous assimilation no longer takes place."

It is evident to me that the author whom I have just cited, has confounded in one description, two distinct maladies. Thus, after having described the form, volume and color of the tumors of the gums, and exposed the numerous evil consequences which they may produce when they have attained a great size and become ulcerated, he adds, "on the upper jaw, these fungi proceed from within outwards from the maxillary sinus," and he gives a description very exact in other respects of these latter. Léveillé has still the error of referring to a single species and an identical origin, all varieties of epulis, and that in spite of the case which proved to him that certain of these tumors are superficial, hard and indolent, and that they may succeed local causes. However that may be, this surgeon has had the merit of dwelling upon the gravest form of epulis, which imperatively demands the intervention of the surgeon.

Richerand hardly mentions this disease. As for Boyer, in his *Treatise upon Surgical Diseases*, he has described only the two simplest forms of epulis. "Some," says he," are smooth and even on their surface ; others have chaps, cracks and ulcerations, whence issues a purulent blood." He says nothing of the composition and intimate structure of these tumors, which he appears to regard as usually benign.

The author of the article *Epulis* in the *Dictionary of Medical Science,* begins by distinguishing the *sclero-sarcoma*, or sarcoma of the maxilla, from the tumors of the gums or *epulis ;* and he establishes afterwards, five varieties of this last malady, viz.

1. Simple epulis, without alteration of the gums.
2. Cartilaginous epulis.
3. Epulis following parulis (gumboil) occasioned by caries of one or several teeth.
4. Epulis with caries of the maxilla.
5. Epulis produced by necrosis of the maxilla.

This classification, altogether arbitrary, does not appear to me justified even by the facts which the author has cited in support of it. In fact, in the first observation, entitled *simple epulis,* there are no hints as to the nature of the tumor, which has not even been taken out. For the third, entitled *cartilaginous epulis,* there is nothing said of the cause which could have given rise to it, and the author is content with saying that the tumor was cartilaginous. Finally, upon the fourth and fifth cases, which I shall presently transcribe, and which are attributed, the one to caries, the other to necrosis of the maxilla, there is absolutely nothing said of the structure of the tumors.

M. Begin, in the *Dictionary of Medicine and Practical Surgery*, appears to admit two distinct varieties of epulis. "The texture of the tumors which we are examining," says he, "is ordinarily soft, spongy, vascular. They swell and harden under the influence of vascular excitation, then subside and lose their volume, when the blood ceases to be attracted to their tissue. At other times, they are hard, fibrous, incompressible, composed of a compact (serré) tissue, in resisting layers interrupted in all directions. I have very recently removed a tumor of this kind, situated above the canine and small molar of the right upper maxilla. It was globular, had the volume of a small nut, and contained in its centre a very compact bony nucleus ; the rest of its substance was a fibro-cartilaginous tissue."

Marjolin and A. Berard, authors of the article *Gums*, (*diseases*,) of the *Dictionary of Medicine*, admit three distinct species. "Some," say they, "are soft, fungous, indolent, they have the greatest analogy with the fungosities which we sometimes see form on the surface of atonic ulcers and of carious bones. They are of an obscure red, and tear and bleed readily, and are ordinarily occasioned by the caries or necrosis of a tooth or of a portion of the alveolar border."

"Other varieties of epulis are of a firmer tissue, more elastic, and of a livelier red ; they subside under compression, and rapidly return upon themselves, when we cease to compress; we feel in them arterial pulsations; these pulsations are even visible. These tumors are covered by the membrane of the gums ; their organization appears to be the same as that of erectile tumors. So long as they are not broken, nothing oozes from them ; if they are cut, they pour forth abundantly a red, vermilion, arterial blood ; they may supervene upon some contusion or appear without any known cause." Finally, we meet with hard epulides, embossed, pale, or of a muddy violet hue, some of which are indolent, while others are the seat of heavy pains or lancinating throbs of greater or less acuteness.

Samuel Cooper exposes the causes of these tumors, the accidents which they occasion, and their treatment, but he expatiates but little upon their structure. "Their tissue," says he, "is generally soft, spongy and vascular ; but sometimes also, it is hard, fibrous, incompressible and endowed with little vascularity." He adds that when they are soft and vascular, they generally spring from the gum itself ; while those of fibrous or fibro-cartilaginous tissue almost always originate in the alveoli.

The facts which we shall presently adduce, will show that this assertion is far from being generally exact, and that Samuel Cooper does not appear to have been acquainted with the gravest of all the species of epulis.

Among the authors of the most modern treatises on external pathology, some say nothing of tumors of the gums, and others content themselves with mentioning them. M. Nelaton alone treats of them more at length ; but the description which he gives of epulis is based upon that of Marjolin and A. Berard, and he admits, without question, the three species described by these authors.

The authors of the new *Dictionary of Nysten*, MM. Littié & Robin, admit in like manner, and describe, almost in the same terms, the three species or varieties of epulis of Marjolin and A. Berard ; they then give important hints upon the pathological anatomy of these tumors, not to be found in the above cited works, and which we consider it our duty to transcribe.

"The study of the structure of the tumors called *epulis*," say they, "shows that under this name are included three species of morbid products :

1. Simple vegetations, or small tumors formed about some carious tooth, &c., composed of amorphous matter, of fibro-plastic elements, and of a web of cellular tissue.

2. Fibrous tumors of the periosteum, whether they invade the bone or not, or sometimes issuing solely from the gum.

3. Those forms of epulis called erectile or embossed, bluish, always invading the maxillary bone to a greater or less extent, and often, the varieties of epulis called cancerous, have for their essential element *myéloplaxes*, a normal element of bone, which is hypertrophied in this case and multiplied; then fibro-plastic elements, medullary cells, fibres of cellular tissue and often numerous vessels.

Having for a fundamental element, a tissue which exists in normal bone, it is not surprising that the tumor when cut away is reproduced. It is this reproduction and the physical characters of the tumor which have caused it to be confounded with cancer. As a general rule, they are not reproduced when the ablation has left no diseased tissue in the bone. It is easily understood that the origin of the disorder being an element of the medulla of bones, these should be invaded by the tumor; but what is specially to be noticed is the resorption of the osseous tissue before the soft tissue during its growth."

II. *Clinical Facts.*—It is easy, on comparing the opinions of the different authors I have just cited, to perceive that science is still far from having settled the clinical and anatomical characters of the different tumors of the gums, united under the common denomination of epulis. In fact, some see but a single disease in different degrees; others admit only two species, the majority recognizing three; and it does not appear doubtful that, if the descriptions furnished by different authors were all correct, one must admit a greater number of varieties.

Desirous to inform myself upon this subject, I have attempted, setting aside the opinions, perhaps preconceived of classic authors, to interpret for myself the language of facts. Consequently, I have devoted myself to long and laborious researches in the periodicals published since the commencement of this century. My hope has almost been disappointed, for I have been able to discover but a very small number of recorded cases of this disease.

These facts amount to ten, half of which alone can be considered as in some measure complete. Adding to them the five related in the *Dictionary of Medical Sciences,* those included in the *Memoirs of the Academy of Surgery,* and one which is found in the writings of Desault, we have a total of twenty-one recorded cases, of which, six at least are absolutely devoid of scientific value. It is with these feeble resources, added to the facts which belong properly to me, that I am about to attempt the determination of the clinical character, and anatomical nature of tumors of the gums.

First of all, it seems necessary strictly to define the limits of my subject.

I give the name of *epulis, épulie,* or *épulide* (from Επι, *upon,* and Οστεο, *gum*) to all fungous, vascular, hard, or carcino matous tumors, which, springing from the gums, from the alveolo-dental periosteum, or from the alveolar arches, are developed in the interior of the mouth. Exostosis, fibrous, cancerous, or other tumors of the lower jaw, are perfectly distinct from these.

Thus limited, the genus *epulis* includes several species which have neither the same characters nor the same importance.

At the head of the group we must place those simple, mamillated, multiple excrescences, of the color and the consistence of the gums, which are formed sufficiently frequently among persons who have a bad denture, or whose gums are subjected to frequent causes of irritation. The following case is an example of simple epulis developed under the influence of a local cause.

CASE I.—*Simple Epulis of the Upper Jaw, caused by a Carious Tooth.* (By the Author.)

M. de S., naval captain, commanding the brig of war *Alcibiade,* was attacked, in the month of June, 1849, with an abscess, situate below the mucous membrane, on the left of the frænum of the upper lip, and caused by an old caries of the first incisor on that side. A puncture with the lancet, giving exit to a small quantity of pus, appeared to accomplish, in a few days, the cure of this abscess. But at the end of fifteen days, there formed on the spot where the puncture had been made, an excrescence of the mucous membrane of the gum, as large as a lentil, which far from disappearing upon cauterization with nitrate of silver, steadily increased. Several times I cut it out with scissors, cauterizing the little wound thus made; nevertheless, it still returned.

Persuaded that this excrescence was caused by the dental caries which often gave M. de S. pain, I advised him to have this tooth extracted; but he refused, and ceased, for a while, to speak to me about his little tumor. Still, having one day interrogated him concerning it, I was astonished to find in the same place, a process of a rosy color, elongated in the form of a rat's tail, and being more than a centimetre in length. No opening was observed; no liquid exuded from it, and its tissue resembled that of the gum.

For more than a year, this excrescence continued to reproduce itself, after having been cut, and M. de S. had it still when I lost sight of him.

The same cause, that is to say, the presence of a carious tooth, or simply of a stump buried in its alveolus, may give rise to fungosities, which being susceptible of acquiring considerable volume, constitute one of the most frequent, although least grave forms of epulis. These fungosities, when they are simple, that is to say, not accompanied by the production of fibrous or cartilaginous elements, may spontaneously disappear after the removal of the cause which produced them; but it is not always so. Besides, it is, sometimes, only after an operation that we can establish the presence of a stump or a bad tooth.

The following case, although leaving something to be desired, may be considered as an example of benign fungous epulis, due to the cause which we are considering. It was undoubtedly, also, the presence of a necrosed tooth which occasioned the formation of an epulis in the subject of case III, although this cause had not been suspected before the operation.

CASE II.—*Epulis of the Upper Jaw, caused by Caries of a Tooth.—Ligature and Cauterization—Cure.* (JOURDAIN.)

M. A. P., Doctor of Medicine, of Paris, recommended to me a woman, about 45 years of age. She had for a long time, upon the gum above the two large upper molars of the right side, an epulis of the size of a large nut, which deformed the cheek. This species of sarcoma covered the last molar, and extended in front over the first bicuspid; but its shortened pedicle was placed directly upon the second molar, which was carious, and which I extracted, causing a hemorrhage that I checked by compression. The fear of a return of the bleeding, should I attempt the excision of the tumor, determined me to have recourse to a ligature, which I tightened daily. On the sixth day the tumor fell; but, as the pedicle had a certain volume, and I had reason to apprehend its return, and perhaps something worse, I thought the actual cautery preferable to all other caustics. After the eschar came off, the bone appeared uncovered, but white and solid, which hindered me from attacking it. I prescribed a vulnerary and detersive gargle, which finished the disease in a short time, without exfoliation of the bone."

It is only from the cause and progress of the tumor that we can suspect a fungous epulis, in fact, nothing in this recital can give us assurance in that respect. What especially interests us is, that the tumor was independent of the maxillary bone which appeared sound on the separation of the eschar.

CASE III.—*Tumor of the Upper Maxilla produced by a Tooth—Extirpation—Cure.* (DR. WERNER GIERGIE, of Milan.)

A young man 18 years of age had, on the upper alveolar margin of the left side, a tumor which had insensibly increased till it occupied half the palatine arch. Fluctuation had been detected and an opening made; laudable pus had escaped, but the tumor had not diminished. The first molar which was loose, had been removed. The patient, having entered the General Hospital of Milan, was placed in Dr. Guecchi's ward. The operation was appointed for the 9th of August, 1838. The cause of the malady, which was supposed to be an exostosis, had not been decided. The second incisor and the second molar was loose. The operation was undertaken in the following manner. The loose teeth were first removed; the cheek was detached from the subjacent tumor; then with a strong, double-edged knife, the bone was divided anteriorly down to the termination of the tumor, which was removed with two blows of the chisel. The maxillary sinus was healthy. The tumor was examined, and in it, to the great astonishment of all the spectators, was found a tooth which was recognized to be the canine. The patient recovered."

There is nothing said, in this report, of the aspect, the color or the consistence of the tumor, the nature of which remains unknown to us. Still, the fact that it followed an abscess and contained a tooth buried in it, leads us to believe that it was simply fungous. After having extirpated the tumor with a cutting instrument, it was not thought necessary to have recourse to cauterization, and nevertheless the tumor does not appear to have been reproduced. It was therefore benign in its character.

In the three preceding cases, the development of the tumors may be reasonably attributed to the presence of carious or necrosed teeth. The patients suffered no pain; at least no mention is made of it, and the cure was prompt and without relapse, in the two cases in which the local cause was removed.—*Revue Therapeutique du Midi.*

**[To be continued.]**

